# Stimulation of *Nicotiana tabacum* L. In Vitro Shoot Growth by Endophytic *Bacillus cereus* Group Bacteria

**DOI:** 10.3390/microorganisms9091893

**Published:** 2021-09-06

**Authors:** Elena Andriūnaitė, Inga Tamošiūnė, Monika Aleksandravičiūtė, Dalia Gelvonauskienė, Jurgita Vinskienė, Rytis Rugienius, Danas Baniulis

**Affiliations:** Institute of Horticulture, Lithuanian Research Centre for Agriculture and Forestry, Kaunas str. 30, Babtai, 54333 Kaunas reg., Lithuania; elena.andriunaite@lammc.lt (E.A.); inga.tamosiune@lammc.lt (I.T.); monikaaleksan@gmail.com (M.A.); dalia.gelvonauskiene@lammc.lt (D.G.); jurgita.vinskiene@lammc.lt (J.V.); rytis.rugienius@lammc.lt (R.R.)

**Keywords:** *Bacillus* sp., culturable endophytic bacteria, microbiome engineering, plant stress

## Abstract

In vitro plant tissue cultures face various unfavorable conditions, such as mechanical damage, osmotic shock, and phytohormone imbalance, which can be detrimental to culture viability, growth efficiency, and genetic stability. Recent studies have revealed a presence of diverse endophytic bacteria, suggesting that engineering of the endophytic microbiome of in vitro plant tissues has the potential to improve their acclimatization and growth. Therefore, the aim of this study was to identify cultivated tobacco (*Nicotiana tabacum* L.) endophytic bacteria isolates that are capable of promoting the biomass accumulation of in vitro tobacco shoots. Forty-five endophytic bacteria isolates were obtained from greenhouse-grown tobacco plant leaves and were assigned to seven *Bacillus* spp. and one *Pseudomonas* sp. based on 16S rRNA or genome sequence data. To evaluate the bacterial effect on in vitro plant growth, tobacco shoots were inoculated with 22 isolates selected from distinct taxonomic groups. Four isolates of *Bacillus cereus* group species *B. toyonensis*, *B. wiedmannii* and *B. mycoides* promoted shoot growth by 11–21%. Furthermore, a contrasting effect on shoot growth was found among several isolates of the same species, suggesting the presence of strain-specific interaction with the plant host. Comparative analysis of genome assemblies was performed on the two closely related *B. toyonensis* isolates with contrasting plant growth-modulating properties. This revealed distinct structures of the genomic regions, including a putative enzyme cluster involved in the biosynthesis of linear azol(in)e-containing peptides and polysaccharides. However, the function of these clusters and their significance in plant-promoting activity remains elusive, and the observed contrasting effects on shoot growth are more likely to result from genomic sequence variations leading to differences in metabolic or gene expression activity. The *Bacillus* spp. isolates with shoot-growth-promoting properties have a potential application in improving the growth of plant tissue cultures in vitro.

## 1. Introduction

Plants are closely associated with endophytes, a group of endosymbiotic bacteria and fungi that live in plant tissues [1,2]. The plant-growth-promoting properties of the endophytes have been extensively studied [3]. The diversity of the cultivated tobacco *(Nicotiana tabacum L.)* endophytic microbiome has been assessed using cultivation-based and 16S rRNA gene metagenomic analysis methods. Sequencing-based analysis revealed that endophytic Enterobacteriaceae communities predominately colonize tobacco seeds with varied abundance among the distinct cultivars; meanwhile, a genotype-specific signature was mainly observed among *Alpha-proteobacteria* [4]. Enterobacteriaceae was also shown to dominate the bacterial community of fresh tobacco leaves, and in terms of abundance it is followed by Pseudomonadaceae, Sphingomonadaceae, Xanthomonadaceae, Moraxellaceae, Bacillaceae, Comamonadaceae and Methylobacteriaceae [5]. *Bacillus* spp., such as *B. pumilus*, *B. amyloliquefaciens*, *B. cereus*, *B. subtilis*, *B. flexus*, *B. simplex* and *B. megaterium*, were dominant among the 11 species isolated from tobacco leaves using a cultivation-based approach [6]. Gao et al. [7] described plant-growth-stimulating properties of phosphate-solubilizing bacteria isolated from the tobacco rhizosphere. However, the plant-growth-promoting potential of tobacco endophytic bacteria has not been assessed to date.

Plant in vitro propagation techniques based on axillary bud proliferation are often applicable in plant biology research, germplasm preservation and the industrial-scale production of vegetatively propagated plants, such as ornamentals, vegetables and agronomic crops [8,9]. Several studies have shown that endophytic bacteria are common in plant tissues grown in vitro [2,10,11] and their composition depends on explant origin and cultivation conditions. Until recently, the composition and role of the endophytic microbiome of in vitro plant organs and tissue cultures attracted little attention and were mainly addressed as contamination resulting from endophytic bacteria overgrowth [11,12,13,14,15]. However, during the last decade, several studies have shown that bacterial endophytes are common in plant tissues grown in vitro and their beneficial effects on plant culture growth, acclimatization and rooting have been recognized. These have been demonstrated for endophytic bacteria isolated from poplar [16], tomato [17], grapevine [18], sweet cherry [19], pineapple [2], purple coneflower [20], strawberry [21] and apple [22] plants.

Endophytic bacteria *Rhodopseudomonas palustris* and *Microbacterium testaceum* co-cultivation experiments with sweet cherry (*Prunus avium)* shoot cultures stimulated the rooting of difficult-to-propagate genotypes [19]. Endophytic *Azospirillum* *brasilense* and *Gluconacetobacter diazotrophicus*, inoculated singularly and together, exerted plant growth-promoting effects on tomato (*Lycopersicon esculentum*) plants grown in vitro [17]. The drought-stress-reducing activity of endophytic *Bacillus* and *Pseudomonas* spp. strains were described in grapevine (*Vitis vinifera*) plants grown in vitro [18]. The effect of six distinct strains of *Pseudomonas* and *Arthrobacter* spp. on growth and metabolite accumulation in purple (*Echinacea purpurea*) and narrow-leaved (*E. angustifolia)* coneflower plants in vitro was described by Maggini et al. [20]. Our previous study showed apple (*Malus* × *domestica*) shoot growth and auxiliary shoot proliferation promoting effect induced by co-cultivation with *Bacillus* spp. or *Pseudomonas fluorescens* strains [22].

The in vitro environment involves a set of conditions, such as the composition of cultivation media, low irradiance, low CO_2_ concentration during the light period and high air humidity, which could lead to the imbalance of plant physiological equilibrium and stress [23,24,25], resulting in slow plant growth and early senescence or severe physiological responses, such as habituation or hyperhydricity [26,27]. Engineering of the endophytic microbiome of in vitro plant tissues has the potential to improve acclimation to stress and improve plant growth under in vitro conditions. Therefore, the aim of this study was to identify *Nicotiana tabacum* endophytic bacteria isolates that are capable of promoting the biomass accumulation of in vitro shoots. Culturable endophytic bacteria were isolated from leaves of greenhouse-grown tobacco plants and their capability to colonize in vitro shoot tissues and promote biomass accumulation was assessed. The taxonomic identity of closely related *Bacillus cereus* group isolates was confirmed using genome sequencing. Functions that could potentially be involved in plant growth-promoting properties were assessed using comparative genomic analysis of the closely genetically related *Bacillus toyonensis* isolates.

## 2. Materials and Methods

### 2.1. Isolation of Cultivable Endophytic Bacteria

Seeds of cultivated tobacco (*Nicotiana tabacum* L.) were planted in non-sterile or autoclaved peat substrate in plastic pots (10 cm × 10 cm) and were grown under greenhouse conditions for one week after seedling emergence.

Fresh tobacco leaves were surface sterilized using a modified protocol described by Zhang et al. [28]. Leaves were thoroughly washed with running tap water, rinsed with 70% ethanol and incubated for 4–5 min in 2.5% sodium hypochlorite. Subsequently, the samples were washed for 30 s with 75% ethanol and rinsed three times with sterile distilled water. The water from the final rinse was plated out on lysogeny broth (LB) agar [29] to confirm that the surface sterilization process was successful. The leaf tissues were mechanically homogenized in sterile deionized water and plated on LB medium. Plates were incubated at room temperature for 2 to 5 days, depending on bacterial colony growth. The bacteria isolates were selected based on distinct colony morphology and further purified by repeated streaking on LB agar. Isolates were grown in LB broth; the medium was supplemented with 25% (*v*/*v*) glycerol and stored at −70 °C.

### 2.2. Identification of Bacterial Isolates and Bacterial Genome Analysis

Bacterial DNA was isolated using the GeneJET Genomic DNA Purification kit (Thermo Fisher Scientific Baltics, Vilnius, Lithuania). The 16S rRNA gene sequence was amplified using the universal primers E8F (5′-AGAGTTTGATCCTGGCTCAG-3′) and E1541R (5′-AAGGAGGTGATCCAGCC-3′) [30]. PCR was conducted using 0.5 µM primer and MyTaqTM Mix 2X (BioLine, London, United Kingdom) under the following conditions: initial denaturation at 94 °C for 2 min, 40 cycles of 94 °C for 30 s, 55 °C for 45 s and 72 °C for 2 min, followed by a final elongation at 72 °C for 10 min. PCR products were purified using the GeneJET PCR Purification kit (Thermo Fisher Scientific Baltics) and sequenced from both ends using the same primers (BaseClear, Leiden, The Netherlands). To reduce bias from sequencing errors, 100 and 20 nucleotides were removed from the beginning and end of the sequences, respectively, and sequences obtained using forward and reverse primers were combined into a single sequence of approximately 1450 bp. The database of the 16S ribosomal RNA sequences (Bacteria and Archea) was queried at the NCBI BLAST server [31] using identity cut-off values of 98.65% for species [32] and 95% for genus [33].

### 2.3. Bacterial Genome Sequencing, Annotation and Comparative Analysis

Whole-genome sequence analysis and assembly were performed by BaseClear using Illumina paired-end sequencing on the MiSeq system. The initial quality assessment was based on data passing the Illumina Chastity filtering, followed by the FASTQC v.0.11.5 process (Babraham Bioinformatics, Cambridge, UK). BayesHammer [34] error-corrected reads were assembled into contigs using SPAdes v.3.10 [35]. Contigs were linked together and placed into scaffolds using SSPACE v.2.3 [36] and gapped regions were partially closed using GapFiller v.1.10 [37]. Assembly errors and nucleotide disagreements were corrected using Pilon v.1.21 [38].

Genome quality and taxonomic analysis were carried out using applications provided by the KBase server [39]. The consistency of the genome assembly was assessed using CheckM v1.0.18 [40]. Average nucleotide identity (ANI) criteria, calculated with FastANI [41] and taxonomic classification of the genome assemblies, was determined using the GTDB-Tk v1.1.0 toolkit [42] and the Genome Taxonomy DataBase (GTDB) release 95 [43], and the Type (Strain) Genome Server (TYGS) search [44]. Genome assembly annotation was performed using the RASTtk annotation engine [45,46]. Gene ontology (GO) terms were assigned using Pannzer2 server [47], summarized using ReviGO server [48] and a semantic similarity plot based on the SimRel measure [49] was built. Analysis of gene clusters encoding the biosynthesis of secondary metabolites and carbohydrate-active enzymes was performed using the bacterial version of the AntiSMASH server v.6 [50] and the dbCAN server [51]. The RFPlasmid server was used to predict plasmid contigs from the bacterial genome assemblies [52].

### 2.4. Tobacco In Vitro Shoot Co-Cultivation with Endophytic Bacteria

A shoot culture of cultivated tobacco was maintained on solid Murashige-Skoog (MS) medium [53], supplemented with 0.75 mg L^−1^ 6-benzylaminopurine, 30 g L^−1^ sucrose and 0.8% agar in a climatic chamber (SANYO Electric Co., Osaka, Japan) at 25 ± 3 °C, under fluorescent lamp illumination at 150 μmol·m^−2^·s^−1^ intensity and with a 16/8 h light/dark photoperiod. After four weeks of cultivation, the shoots were transferred to a fresh medium and were used for bacterial inoculation the next day.

Shoot inoculation experiments were carried out as described previously by Tamošiūnė et al. [22]. Bacterial inoculum initiated from a glycerol stock was grown in LB broth at 25 °C to an exponential growth phase. Bacteria were sedimented via centrifugation and resuspended in MS medium at a concentration of ~10^7^ CFU/mL. Three microliters of the bacterial suspension were inoculated on several nodes of the shoot petiole. MS medium without bacteria was used for the control treatment. The inoculated shoots were maintained as described above and shoot fresh weight (FW) was assessed after 3 weeks of co-cultivation. The shoot and endophytic bacteria co-cultivation experiments were carried out two to four times and the mean values of FW were estimated using 30 to 125 shoot samples.

### 2.5. Analysis of Endophytic Bacteria Density in Tobacco Shoot Tissues

Since no bacterial growth was detectable on LB agar for the control shoot extracts, long-term survival of endophytic bacteria in shoot tissues was confirmed and their density was estimated using serial dilution. The inoculated shoots were transferred to a fresh medium every 4 weeks and the density of the inoculated bacteria was assessed after 3 passages. A hundred milligrams of the pooled shoot sample was homogenized in 1 mL LB medium, diluted via serial dilution and plated on LB agar. Two replicates were used for each dilution and the experiment was repeated at least twice. The bacterial isolate identity was confirmed by 16S rRNA gene sequencing as described above.

## 3. Results

### 3.1. Isolation and Identification of Cultivable Endophytic Bacteria

**Figure 1:**
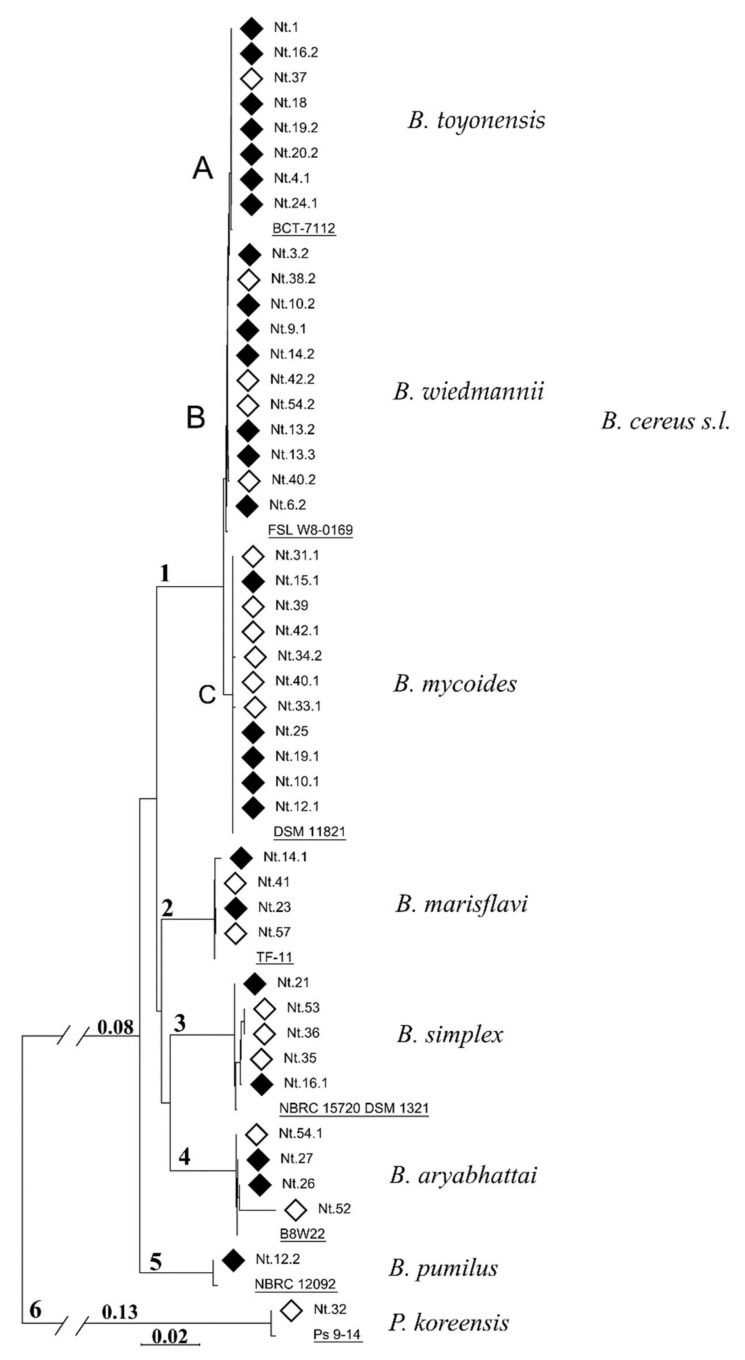
Phylogenetic relationship of endophytic bacteria isolated from common tobacco leaves. The tree was built using a neighbor-joining algorithm [54] using trimmed 16S rRNA gene sequences. The isolates were assigned to six distinct phylogenetic clades (1–6), and clade 1, representing *B. cereus s.l.*, was further divided into three subclades (A–C) according to the taxonomic identity. The taxonomic assignment of the phylogenetic clusters was based on the reference strains (underlined), that were obtained either through the querying of the genome data of representative isolates (Nt.18, Nt.37 and Nt.3.2) in the GTDB and TYGS databases for clusters 1A–B (Table A2) or using an NCBI BLAST search for the remaining clusters (Table A1). The scale bar represents the relative phylogenetic distance. Bacteria isolated from leaves of tobacco grown on non-sterile and autoclaved peat substrate are indicated by black and white diamonds, respectively.

A total of 45 bacterial isolates with distinct colony morphology were obtained from fresh leaves of tobacco grown in a greenhouse on peat substrate for one week. Isolates were obtained from plants grown on both non-sterile and autoclaved peat substrate variants. Based on 16S rRNA gene sequencing analysis, isolates were assigned to six distinct phylogenetic clades (Figure 1; Appendix A, Table A1). Isolates of the largest clade 1 were closely related to *Bacillus cereus sensu lato* (*s.l.*), also known as the *B. cereus* group. The remaining isolates were assigned to other four *Bacillus* species (clades 2 to 6), including *B. marisflavi*, *B. aryabhattai*, *B. pumilus* and *B. simplex*, and one isolate was identified as *Pseudomonas koreensis* (clade 6).

The low variability of the 16S rRNA gene sequence and the similar colonial morphology within closely related subclades 1A and 1B led to unequivocal identification results. The closest match for the 16S rRNA sequence-based BLAST search included *B. thuringiensis* for the isolates of subclade 1A and a variation of *B. mobilis* and *B. wiedmannii* for subclade 1B. Through the taxonomic analysis of the genome data of the representative isolates (Nt.18, Nt.37 and Nt.3.2), we assigned clusters 1A and 1B to the species *B. toyonensis* (ANI > 99.3%) and *B. wiedmannii* (ANI > 96.5%), respectively (Appendix A, Table A2). The ANI value between the two genomes within cluster 1A was approximately 99.5%; meanwhile, the ANI estimate between the genomes of the two discrete clusters was 91.4% (Appendix A, Table A3), which would be below the 95% demarcation threshold for species.

### 3.2. Endophytic Bacteria Co-Cultivation Effect on Tobacco Shoot Biomass Accumulation

**Figure 2:**
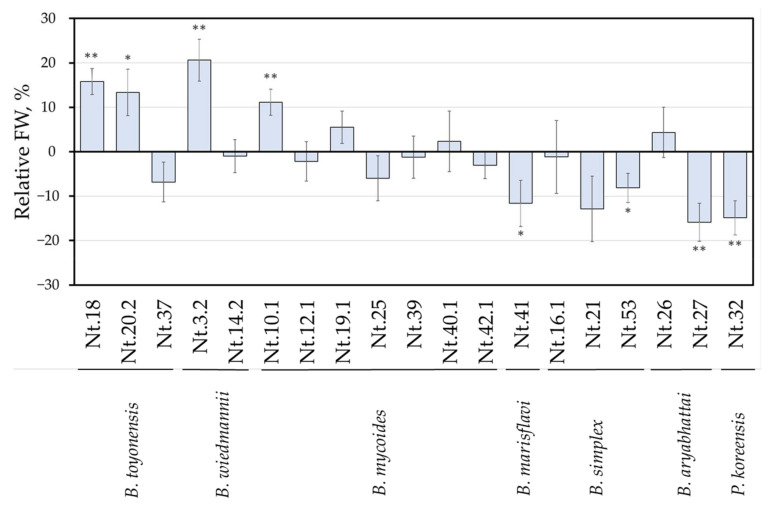
Endophytic bacteria co-cultivation effects on tobacco shoot biomass accumulation. Data are presented as the mean and standard error of the mean of co-cultivated tobacco shoot fresh weight (FW) normalized to controls. The asterisks indicate mean values that were significantly different compared to controls (* *p* < 0.05; ** *p* < 0.01).

**Figure A1:**
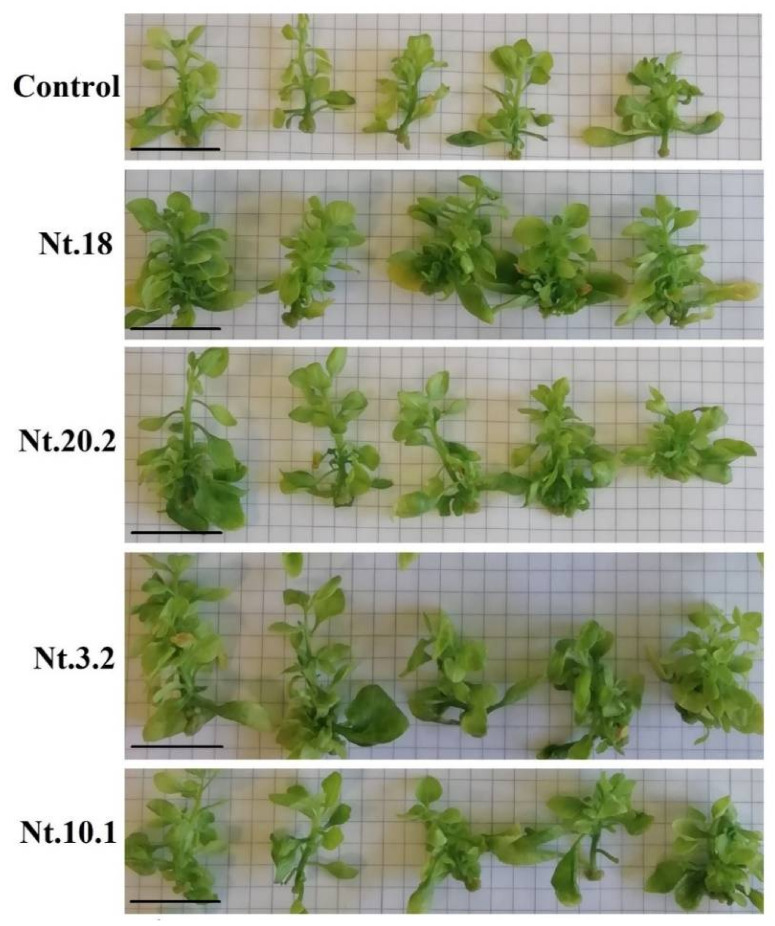
Tobacco (*Nicotiana tabacum* L.) shoots after 3 weeks of co-cultivation with isolates *Bacillus toyonensis* Nt.18 and Nt.20.2, *Bacillus wiedmannii* Nt.3.2 and *Bacillus mycoides* Nt.10.1. Isolates had a significant growth-promoting effect (11% to 21% increase in shoot biomass) as compared to control shoots. Shoots were inoculated with 3 µL of each strain suspended at ~10^7^ CFU/mL in MS medium. MS medium without bacteria was used for the control treatment. Five representative shoots are shown for each experimental group. Scale bar represents 2 cm.

To evaluate the effects of bacterial isolates on plant growth in vitro, tobacco shoots were inoculated with a suspension of bacteria. Shoot FW was assessed after 3 weeks of co-cultivation and the analysis included a representative set of 22 isolates selected from distinct taxonomic groups. Inoculation with isolates Nt.9.1, Nt.12.2 and Nt.54.1 resulted in a detrimental effect on shoot viability. Co-cultivation with the isolates of *B. aryabhattai*, *B. marisflavi*, *B. simplex* and *P. koreensis* either had no significant effect on biomass accumulation or the shoot growth was reduced (Figure 2). Meanwhile, isolates belonging to the *B. cereus s.l.* group were the most effective in promoting tobacco shoot biomass accumulation. Among the thirteen tested isolates of the *B. cereus* group, four resulted in an 11% to 21% increase in shoot biomass accumulation as compared to control shoots (Appendix A, Figure A1).

Interestingly, co-cultivation with closely related isolates within the *B. cereus s.l*. group resulted in a contrasting effect on shoot growth, as can be seen in Figure 2*. B. wiedmannii* Nt.3.2 promoted shoot growth (21 ± 4.7% FW increase as compared to control); whereas no effect was detected for the isolate Nt.14.2. Similarly, *B. toyonensis* Nt.18 and Nt.20.2 stimulated a 16 ± 2.9% and 13 ± 5.2% increase in shoot biomass, respectively, but a shoot-growth-inhibiting trend was observed for the Nt.37 isolate. In addition, among the seven *B. mycoides* isolates included in the analysis, significant shoot-growth-promoting properties (11 ± 2.9%) were observed only for Nt.10.1.

### 3.3. Survival of Endophytic Bacteria Isolates in Tobacco Shoot Tissues

Colonization and survival in plant tissue is an essential property of endophytic bacteria. Therefore, the survival of the bacterial isolates in tobacco shoot tissues in vitro during the extended co-cultivation period, corresponding to three passages onto a fresh medium, was assessed using a serial dilution and plating approach. It was estimated that shoots co-cultivated with *B. toyonensis* isolates Nt.18 and Nt.37 had very similar bacterial cell density, corresponding to 6 ± 2 × 10^5^ and 5 ± 2 × 10^5^ CFU/g FW, respectively. Meanwhile, significant variations in bacterial density were observed for *B. wiedmannii* isolates Nt.3.2 and Nt.14.2 (1.7 ± 0.3 × 10^5^ and 11 ± 3 × 10^5^ CFU/g FW, respectively; *p* = 0.013).

### 3.4. Comparative Genome Analysis of Closely Related *B. toyonensis* Isolates

**Figure 3:**
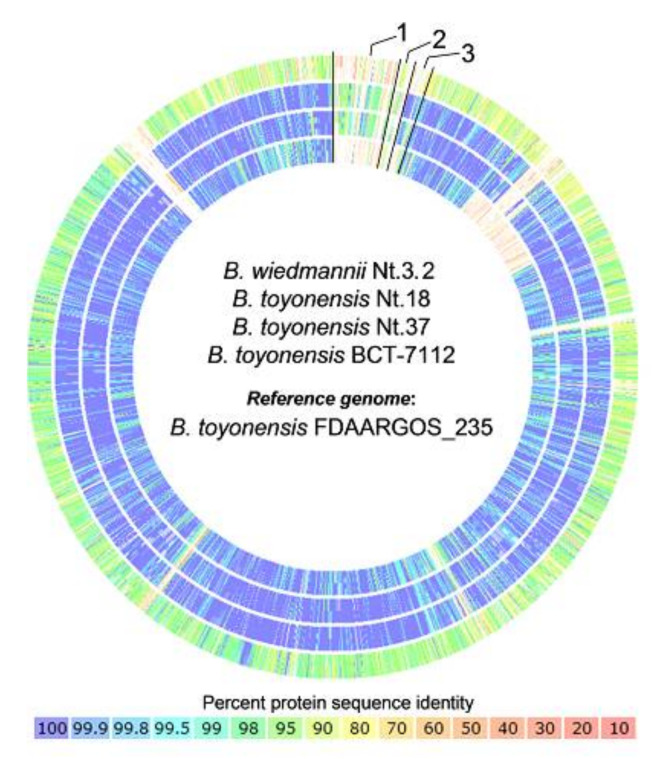
Protein sequence-based comparison of genome data of *B. toyonensis* isolates Nt.18 and Nt.37, strain BCT-7112 used as a *B. toyonensis* species representative and an isolate of the closely related species *B. wiedmannii*, Nt.3.2, with reference genome of strain FDAARGOS_235 (not shown). Numbers indicate segments corresponding to three plasmids identified in the reference genome. The color represents the percentage of sequence identity.

Whole-genome sequencing of the two *B. toyonensis* isolates Nt.18 and Nt.37 with contrasting shoot-growth-modulating properties provided a genomic assembly of higher than 99.3% completeness (Appendix A, Table A2) and a closely related genomic sequence overall, with ANI defined at ~99.5% between the two isolates (Appendix A, Table A3). Therefore, comparative genomics analysis was used to assess the intraspecific differences between the two genome assemblies to identify features potentially linked to the growth-promoting properties of the isolate Nt.18. Through the subsystem-based annotation procedure [45,46], we identified 5761 and 5814 coding genes for the Nt.18 and Nt.37 genome assemblies, respectively (Appendix A, Table A2), including 63% of non-hypothetical proteins. For protein sequence-based comparison, *B. toyonensis* FDAARGOS_235 (RefSeq accession GCF_002073415.2), with a complete genome sequence assembly, was used as a reference organism. In addition, strain BCT-7112 (GTDB identifier GCF_000496285.1) was used as a *B. toyonensis* species representative in the GTDB and a representative isolate Nt.3.2 of the closely related *B. wiedmannii* species was included in the analysis (Figure 3; Supplementary Materials Table S1). The analysis revealed that *B. toyonensis* Nt.18 and Nt.37 shared 72% and 75% identical protein sequences with the reference organism and 71% between themselves, whereas ~93% of sequences had ≥90% identity for all of the organisms.

Visualization of the protein sequence-based comparison shows several low-homology segments (Figure 3). The largest segment is related to the region identified as two of the three plasmids in the genome assembly of *B. toyonensis* FDAARGOS_235. The first plasmid shows a partial match between the isolates Nt.18 and Nt.37, meanwhile the second appears completely absent in the genome assemblies. Several other low-homology regions have a similar distribution in all genomes used for the comparison, suggesting the presence of unique features in the chromosome of the reference organism or possibly common inaccuracies of the short-read WGS assemblies.

**Figure 4:**
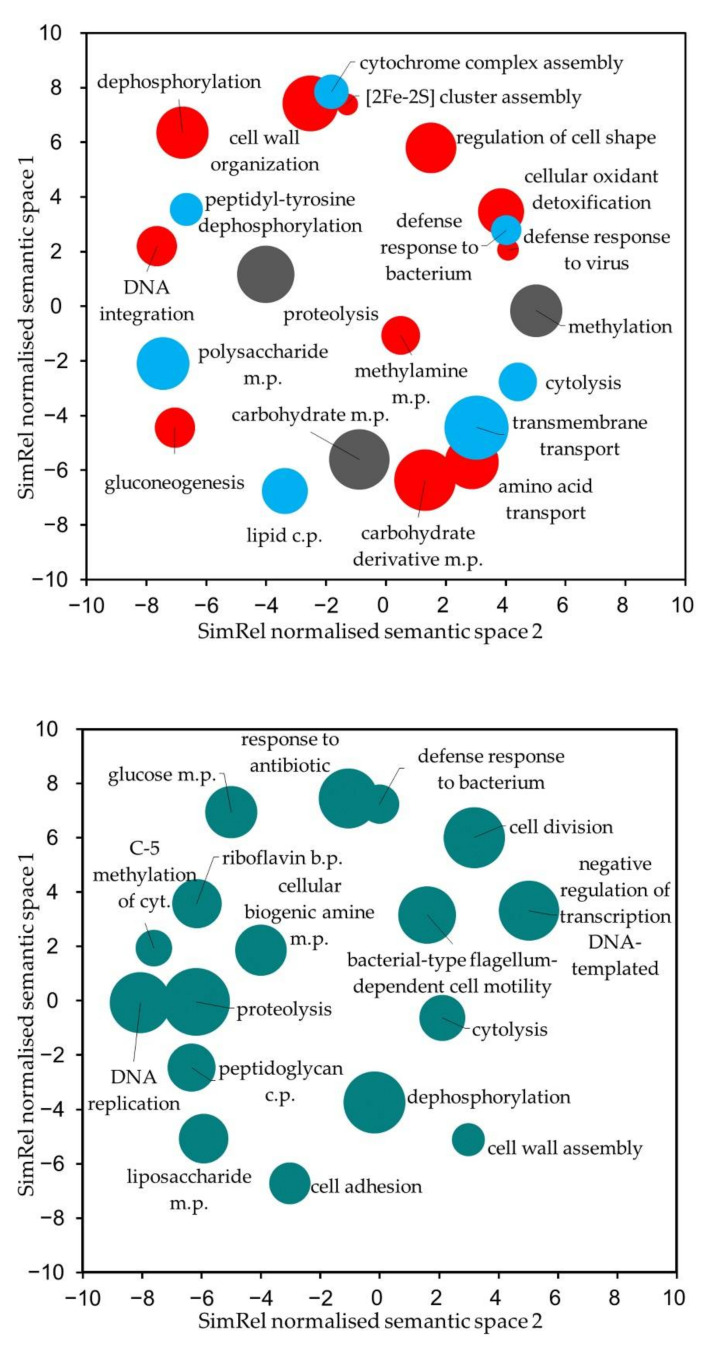
GO term semantic relationship-based visualization of distinct functional features of *B. toyonensis* isolates Nt.18 and Nt.37 identified by protein sequence-based genome comparative analysis. GO terms of singleton features (**A**) and a set of low-amino-acid-identity proteins (<90%) of core features (**B**) were summarized using the ReviGO algorithm. Bubble size indicates the log value of the frequency of the GO term in the GO annotation database (bubbles of more general terms are larger). The red, blue and gray colors of the bubbles correspond to GO terms that were unique to isolates Nt.18 and Nt.37 or common to both isolates, respectively. Abbreviations: c.p.—catabolic process, m.p.—metabolic process, cyt.—cytosine, b.p.—biosynthetic process.

To investigate features that could be related to the growth-promoting properties of the isolate Nt.18, we began our analysis with a pairwise protein sequence-based comparison of the two *B. toyonensis* genome assemblies, which revealed 185 and 220 singleton features unique either to Nt.18 or Nt.37, respectively (Appendix A, Table A4; Supplementary Materials Tables S2 and S3). In addition, 151 sequences with <90% identity between the two genomes were included in the further analysis based on the assumption that low sequence homology could also potentially lead to a variation in metabolic or signaling functions that could be related to the growth-promoting properties. After the sequences of unidentified and hypothetical proteins and sequences related to phage and mobile elements were excluded, the three sets included 71, 20 and 52 features, respectively (features and identified GO terms are provided in Supplementary Materials Table S4). Based on the analysis of the biological process GO terms, genes unique to the genome of Nt.18 were mainly involved in nucleic acid, protein and carbohydrate metabolic processes, as well as processes related to the stress response, such as cellular oxidant detoxification or the defence response to viruse (shown as red bubbles in Figure 4A). Meanwhile, genes unique to the Nt.37 genome were mainly related to protein, carbohydrate and lipid biosynthesis, but also included the processes of cytolysis, cell wall formation and the defence response (blue bubbles in Figure 4A). Similarly, among the proteins with sequence identity <90% the prevalent biological process was related not only to nucleic acid and protein metabolism (Figure 4B) but also included a variety of other functions related to cytolysis, cell division, cell adhesion, lipids and glucose metabolic or peptidoglycan catabolic processes.

Our further analysis focused on identifying genomic regions encoding enzymes involved in secondary metabolite biosynthesis, and revealed 36 complete or partial biosynthetic gene clusters (BGCs) (Supplementary Materials Table S5), most of which showed high sequence homology between the two genome assemblies. Enzyme composition or sequence homology differences were detected only for a linear azol(in)e-containing peptide (LAP) synthesis cluster and one of the 22 identified saccharide synthesis clusters, with a partial match to an exopolysaccharide (EPS) biosynthesis operon (clusters No. 36 and 20, respectively). The results of the saccharide synthesis clusters were further confirmed using the carbohydrate enzyme analysis server dbCAN, where among the 91 and 95 motifs identified for the isolates Nt.18 and Nt.37, respectively, 86 were shared by both genome assemblies and the remaining unique features corresponded to the identified EPS-related biosynthesis cluster.

**Figure A2:**
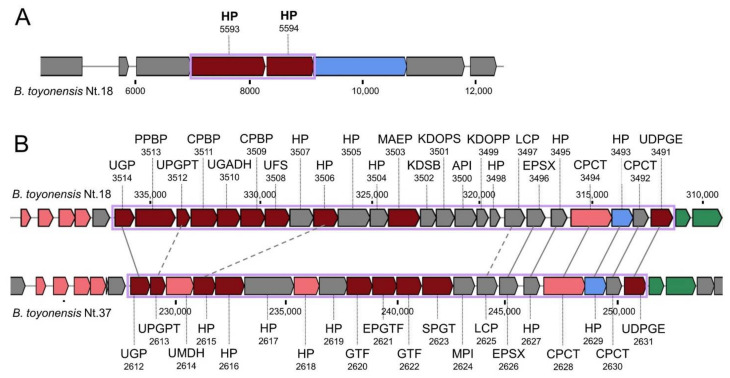
Structure of secondary metabolite biosynthetic gene clusters No. 36 LAP (**A**) and No. 20 EPS related (**B**), identified using the antiSMASH server. Text and number for the CDS labels correspond to protein symbol as indicated in Appendix A, Table A5, and feature number as indicated in Supplementary Materials Tables S1 and S2.

LAP cluster No. 36 included putative cyclodehydratase and nitroreductase enzymes potentially involved in the biosynthesis of the linear azol(in)e-containing peptide (LAP) (Appendix A Figure A2A, Appendix A Table A5). The cluster was located on scaffold 27, which, together with scaffold 26, constituted a ~45 kbp fragment unique to the genome assembly of the isolate Nt.18. Plasmid prediction analysis, using the *Bacillus* sp.-optimized database in the RFPlasmid tool, showed the high plasmid assignment probability of the two scaffolds (0.87 and 0.94 for scaffolds 26 and 27, respectively), suggesting that they represent a complete or partial sequence of the plasmid that is unique to the growth-promoting isolate of *B. toyonensis*.

In contrast, the enzymes encoded in the EPS-related cluster No. 20 were partially shared between the two *B. toyonensis* isolates and were located on scaffolds 4 and 5 of Nt.18 and Nt.37, respectively. The two scaffolds shared a high probability of assignment to the chromosome (0.997), and also included another three clusters of saccharide and betalactone biosynthesis (Appendix A Table A5, cluster No. 7, 21 and 22) that were highly homologous between the two organisms. Among the 12 core or related biosynthesis enzymes encoded by the EPS-related cluster, No. 20, several were partially shared by the two genome assemblies.

The genes located at both ends of the cluster showed high sequence homology between the two organisms (Appendix A Figure A2B, Appendix A Table A5). These included core enzymes involved in EPS biosynthesis, such as UTP--glucose-1-phosphate uridylyltransferase (UGP), cell envelope-associated transcriptional attenuator LytR-CpsA-Psr (LCP), choline-phosphate cytidylyltransferase (CPCT) and UDP-glucose 4-epimerase (UDPGE). The remaining unique sequences of isolate Nt.37 were also mostly related to EPS biosynthesis enzymes, such as UDP-N-acetyl-D-mannosamine dehydrogenase (UMDH), exopolysaccharide biosynthesis glycosyltransferase (EPGTF) or glycosyltransferase (GTF). Meanwhile, the cluster of the Nt.18 genome assembly included unique sequences encoding core enzymes of the lipopolysaccharide (LPS) biosynthesis pathway, such as UDP-N-acetylglucosamine 4,6-dehydratase (UGADH); UDP-N-acetyl-L-fucosamine synthase (UFS), a protein similar to capsular polysaccharide synthesis enzyme Cap5F (CPBP) and several other related enzymes, as well as multi antimicrobial extrusion protein (MAEP).

It is notable that, on a solid medium, the two isolates formed identical white colonies with smooth surfaces. Since variation in EPS biosynthetic activity is largely related to the formation of distinct colony morphologies [55], the EPS-related cluster No. 20 may not be directly involved in EPS biosynthesis. Enzymes homologous to the *Bacillus subtilis* enzymes involved in EPS operon function [56] were detected in other saccharide biosynthesis-related clusters, such as No. 14 and 17 (Supplementary Materials Tables S1 and S5), which included enzymes that are homologous between the two isolates.

## 4. Discussion

The *Bacillus* genus contains over 260 named species of bacteria, including one of the earliest bacteria to be described [57], which are widely spread in the environment and are most readily cultured on basal microbiological media such as LB or nutrient agar. A very sturdy endospore structure enables *Bacillus* species to survive under extremely harsh environmental conditions [58]. Furthermore, the *Bacillus* genus includes several common endophytic bacteria species that provides plants with a wide range of benefits such as increased biological nitrogen fixation, phosphate solubilization and the production of siderophores and phytohormones [59,60,61,62]. In the context of the described properties, it appears reasonable that *Bacillus* spp. represent the majority of bacteria isolated from tobacco leaves in our study. A similar domination of *Bacillus* sp. among the culturable endophytic bacteria isolated from tobacco leaves has been previously described by Chen et al. [6]. Such cultivation-based endophytic bacteria isolation does not reflect the complexity of the tobacco bacterial endophytome revealed by metagenomic analysis [5]. It is likely that the limits of cultivation-based isolation are set by the inability of unculturable bacteria to grow on artificial media in combination with the harsh treatment required for leaf surface sterilization, which likely leads to the excessive disinfection of microorganisms residing in soft tobacco leaf tissues. However, this approach provides a reasonable depiction of the endospore-forming fraction of the bacterial population, which has long been regarded as the most suitable for agricultural probiotic formulations due to its capability of prolonged survival under unfavorable conditions [62,63] as well as for applications based on vertical transmission through seeds [64].

The *B. cereus s.l.* taxonomic group is a subdivision of the genus *Bacillus* that includes closely related species with conserved genomes (5.2 to 5.9 Mb) sharing over 97% similarity with the known species of this group (>99% in 16S rRNA gene sequences) and less than 95% similarity with other species of the genus *Bacillus*. In our study, 16S rRNA sequence data and characteristic rhizoidal colonial morphology supported the relation of subclade 1C to *B. mycoides*, which is a genetically distantly related branch of the *B. cereus* group [65]. Meanwhile, the best hit for the 16S rRNA sequence-based BLAST search included *B. thuringiensis* for the isolates of subclade 1A and a variation of *B. mobilis* and *B. wiedmannii* for subclade 1B. Likewise, Carroll et al. [66] have emphasized the difficulty in the reliable differentiation of *B. mobilis* and *B. wiedmannii* strains, which produced overlapping genomospecies in which genomes could share more than 95% ANI for both species. In addition, *Bacillus* sp. strain MC28, previously described as *B. thuringiensis,* was specified as a novel species of the *B. cereus* group [67] based on analysis of phenotypic and genotypic traits, and was clustered into the *B. toyonensis* group [68]. In our study, the analysis of the genome data of the representative isolates assigned clusters 1A and 1B to species *B. toyonensis* and *B. wiedmannii*, respectively.

The vertical transmission of *Bacillus* spp. through plant seeds has been described for switchgrass [69], wheat [70] and tomato [71]. In our study, for all of the species represented by more than one isolate (Figure 1, clades 1–4), isolates were obtained from tobacco plants grown on both substrate variants—non-sterile or autoclaved peat—suggesting that these seedling-colonizing bacteria were either transmitted vertically through tobacco seeds or were inoculated from water or greenhouse environments. Only for *B. toyonensis,* the majority of isolates (seven out of eight) were obtained from plants grown on non-sterile substrates, implying that an origin in the colonization of seedlings from the rhizosphere is more likely. However, this should be confirmed through a more detailed analysis.

Screening for capability to promote the biomass accumulation of in vitro tobacco shoots revealed that the stimulating effect was induced by co-cultivation with four isolates, identified as *B. toyonensis, B. wiedmannii* and *B. mycoides* (Figure 2). These species were previously shown to include plant-growth-promoting strains of endophytic or soil bacteria. Endophytic *B. toyonensis* bacteria were shown to enhance the growth of blueberry and tomato [72,73] and act as an agent for the biocontrol of plant pathogens [73,74,75]. A relatively newly described species of *B. wiedmannii* [76] was found to be a plant-growth-promoting bacteria, isolated from the rhizosphere [77]. Growth-promoting properties of *B. mycoides* have been shown in greenhouse experiments and field studies with wheat [78], rice [79] and sunflower plants [80]. Additionally, it was found that a consortium of *B. mycoides* with other endophytic microorganisms had a beneficial effect on the growth of strawberry plants [81] and maize [82].

Previously *B. pumilus* was shown to enhance the root development of in vitro grapes [83], as well as to promote the growth of seedlings of red pepper [84] and quailbush [85]. Beneficial effects on the growth of tomato [86], corn and soybean [87] by *B. simplex,* and that of wheat seedlings by *Pseudomonas koreensis* [88] have been reported. Furthermore, the plant growth-promoting properties of *B. aryabhattai* strains isolated from various environments have been reviewed by Bhattacharyya et al. [89]. However, seven isolates of the *Bacillus* and *Pseudomonas* species tested in our study had no significant positive effect on the biomass accumulation of tobacco shoots in vitro. The difference might result from bacterial strain- and plant genotype-specific interactions or in vitro cultivation conditions.

The analysis of bacterial isolate survival over an extended co-cultivation period suggested that the absence of a shoot growth-stimulating effect with the isolates Nt.37 and Nt.14.2 was not related to the inability of the bacteria to survive in shoot tissues as their cell density was similar or higher compared to the growth-stimulating isolates. Following three passages on fresh MS medium, the bacterial density in tobacco shoot tissues was within the range typically observed for endophytic bacteria (~6 × 10^5^ CFU/g FW) [90,91,92,93].

Notably, our study revealed a strain-specific intraspecies variation of the tobacco shoot growth-modulating property among the taxonomically related isolates. For example, no significant effect on shoot growth was observed for *B. toyonensis* Nt.37, *B. wiedmannii* Nt.14.2 or for a majority of the tested *B. mycoides* isolates, in contrast to the growth-promoting effect of *B. toyonensis* Nt.18 and Nt.20.2, *B. wiedmannii* Nt.3.2, and *B. mycoides* Nt.10 (Figure 2). Moreover, *B. wiedmannii* Nt.9.1 had a detrimental effect on shoot survival.

The plant stress-reducing and growth-promoting activity of microorganisms is mainly attributed to nitrogen fixation or ACC-deaminase activity, as well as the production of bioactive substances such as siderophores, phytohormones or other secondary metabolites [94,95,96]. In our study, the comparative genomic analysis revealed a set of genes that are unique or share low homology between the *B. toyonensis* isolates Nt.18 and Nt.37. Based on GO analysis, their function was mainly assigned to primary metabolic processes related to DNA, protein or lipid synthesis or modification, as well as processes involved in transport, cell development and the response to stress (Figure 4). This also included several biological processes, such as polysaccharide, carbohydrate or carbohydrate derivative, peptidoglycan or biogenic amine metabolic process, that potentially could be directly involved in secondary metabolite production. Prediction of the gene clusters involved in secondary metabolite biosynthesis revealed a putative enzyme cluster involved in LAP biosynthesis which was unique to the isolate Nt.18 and the EPS biosynthesis cluster partially shared between the two *B. toyonensis* isolates.

The production of azol(in)e-containing peptides has previously been studied mainly in cyanobacteria [97] and *Escherichia coli* [98] and in species of the *Bacillus* genus [99]. The study by Zhao et al. [100] reviewed a distribution of a total of 117 putative gene clusters of LAPs in more than 20 species of Bacillales. However, more detailed experimental studies were carried out and the antimicrobial activity was analyzed only for the plantazolicin produced by *B. amyloliquefaciens* [101,102]. Therefore, the function and potential antimicrobial activity of the putative LAP biosynthesis enzyme cluster No. 36 (Supplementary Table S5) identified in the genome assembly of isolate Nt.18 remains elusive and further investigation is required to establish its significance for antagonistic microbial interactions and plant-growth-regulating activity.

Bacterial production of EPS plays an important role in biofilm formation and plant microbial interactions [103]. EPS biosynthesis, controlled by 16 genes of the *eps* operon, was extensively studied in *Bacillus subtilis* [56,104]. Our analysis revealed several enzyme clusters involved in saccharide biosynthesis in the genome assemblies of the two *B. toyonensis* isolates. One of the clusters (Supplementary Table S5, cluster No. 20), unique to the isolate Nt.18, included a set of enzymes related to LPS biosynthesis. Since Gram-negative bacteria do not produce LPS, the origin and function of the genes coding the enzymes involved in LPS biosynthesis are inexplicable. Previously, a regulatory role for arabinose-5-phosphate, produced by the putative API enzyme identified in the Gram-positive *Clostridium tetani*, was described [105]. However, the authors note that the bioinformatics analysis showed the presence of homolog sequences encoding other enzymes in the LPS biosynthetic pathway in genomes of Gram-positive bacteria, which they speculate to be a result of contamination by Gram-negative bacteria. Considering this and the fact that the disparity within the EPS-related cluster No. 20 of the *B. toyonensis* isolates does not result in morphological changes of bacterial colonies, its role in EPS production or other related functions important for the plant-growth-promoting activity of the bacterium is doubtful.

A similar genomic structure and a lack of concrete genomic evidence for discrete secondary metabolite biosynthesis activity imply that the distinct shoot growth-modulating properties of the two *B. toyonensis* isolates are more likely to result from the genomic sequence variations leading to gene expression and metabolic activity differences. Therefore, further and more detailed genomic analysis would be useful to pinpoint genomic sequence disparities and transcriptome analysis would help to assess variations in gene expression that could be associated with variations in signaling processes and/or the activity of the metabolic pathways important for the plant-growth-promoting properties.

## 5. Conclusions

Our study showed that endophytic bacteria of the *Bacillus cereus* group isolated from cultivated tobacco leaves could promote tobacco shoot biomass accumulation under in vitro conditions. The contrasting shoot-growth-regulating properties observed for closely related isolates of the same species, such as *B. toyonensis*, *B. wiedmannii* and *B. mycoides,* suggested the presence of strain-specific interaction with the plant host. The absence of structural genomic evidence for discrete secondary metabolite biosynthesis by the two closely related *B. toyonensis* isolates suggested that distinct growth-modulating properties would be more likely related to variations in gene expression leading to distinct metabolic activity. This study paves the way for a better understanding of the interaction of *Bacillus cereus* group endophytic bacteria with plant hosts, and bacterial isolates have a potential application in improving the growth of plant tissue cultures in vitro.

## Data Availability

Data is contained within the article or Supplementary Materials.

## Supplementary Materials

The following are available online at https://www.mdpi.com/article/10.3390/microorganisms9091893/s1, Table S1: Protein-sequence-based comparison of genome assemblies of *B. toyonensis* isolates Nt.18 and Nt.37, *B. toyonensis* species reference genomes (strains BCT-7114 and FDAARGO_235) and *B. wiedmannii* Nt.3.2, Table S2: Pairwise protein-sequence-based comparison of *B. toyonensis* isolates Nt.18 and Nt.37 genomic assemblies, Table S3: *Bacillus toyonensis* Nt.18 and Nt.37 singleton features and core feature proteins with amino acid sequence identity <90%, identified using the RASTtk annotation engine, Table S4: GO terms of *Bacillus toyonensis* Nt.18 and Nt.37 singleton features and core feature proteins with amino acid sequence identity <90%, assigned using the Pannzer2 server, Table S5: Biosynthetic gene clusters (BGCs) identified in genome assemblies of the closely related *B. toyonensis* isolates Nt.18 and Nt.37 using antiSMASH.

## Appendix A

**Table A1:** Results of bacterial isolate identification using 16S rRNA gene sequence data.

Clade No. ^a^	Bacterial Strain	Accession ^b^	Accession Length, bp	Sequence Identity, %	Reference
1 A	*Bacillus thuringiensis* IAM 12077	NR_043403.1	1486	97.3–99.9	[106]
1 B	*Bacillus mobilis* MCCC 1A05942	NR_157731.1	1509	97.1–99.8	[107]
1 C	*Bacillus mycoides* DSM 11821	NR_024697.1	1531	97.8–100.0	[108]
2	*Bacillus marisflavi* TF-11	NR_025240.1	1506	98.1–98.9	[109]
3	* Bacillus simplex * NBRC 15720 = DSM 1321	NR_112726.1	1476	95.4–99.7	d.s.
4	*Bacillus aryabhattai* B8W22	NR_115953.1	1533	95.0–97.5	[110]
5	*Bacillus pumilus* NBRC 12092	NR_112637.1	1474	99.89	d.s.
6	*Pseudomonas koreensis* Ps 9-14	NR_025228.1	1455	99.5	[111]

16S rRNA gene sequences were prepared and analyzed using the NCBI BLAST server as described in Materials and Methods. ^a^ Clade numbers as indicated in Figure 1; ^b^ NCBI nucleotide database accession ID; Abbreviations: d.s.—direct submission.

**Table A2:** Statistics of the bacterial isolate genome sequencing, de novo assembly, genome quality assessment, results of taxonomic assignment and annotation.

Parameter	Isolate		
	Nt.18	Nt.37	Nt.3.2
Illumina demultiplexing and read mapping statistics			
Number of read pairs	2,468,194	2,298,751	2,950,725
Yield (mbp)	666	596	797
Average quality	36.03	36.23	36.14
Total reads	4,936,388	4,597,502	5,901,450
Mapped reads	4,899,758	4,571,302	5,869,378
Average coverage	119.6	107.16	135.82
Insert size median	317	272	300
*De novo* short-read assembly statistics			
Genome Length (bp)	5,574,424	5,586,422	5,891,672
GC Content (%)	35.0626	35.090523	34.933907
Contigs N50 (bp)	564,507	276,397	324,644
Number of scaffolds	52	90	91
Average scaffold size (bp)	107,200	62,071	64,743
Max scaffold size (bp)	1,307,898	934,026	1,427,494
Min scaffold size (bp)	311	587	335
Number of gaps	3	5	5
Genome quality statistics			
Completeness (%)	99.34	99.43	99.34
Contamination (%)	0.23	0.11	0.1
Closest placement taxonomic assignment			
Species	*Bacillus toyonensis*	*Bacillus toyonensis*	*Bacillus wiedmannii*
GTDB species representative	BCT-7112	BCT-7112	FSL W8-0169
GTDB reference	GCF_000496285.1	GCF_000496285.1	GCF_001583695.1
TYGS accession	NCIMB 14858	NCIMB 14858	FSL W8-0169
ANI (%)	99.39	99.33	96.49
Alignment fraction (%)	0.95	0.95	0.91
Annotation statistics			
Features	5853	5905	6424
Coding gene	5761	5814	6217
RNAs	92	91	96
Distinct functions	4049	4072	4136
Non-hypothetical proteins	3707	3729	3933
Hypothetical proteins	2054	2085	2284

**Table A3:** Estimates of ANI values between *B. toyonensis* and *B. wiedmannii* isolate genomes using FastANI.

Query	Reference	ANI Estimate	Matches	Total
Nt.18	Nt.37	99.554	1740	1833
Nt.37	Nt.18	99.482	1746	1814
Nt.18	Nt.3.2	91.356	1527	1833
Nt.37	Nt.3.2	91.328	1529	1814
Nt.3.2	Nt.18	91.242	1553	1923
Nt.3.2	Nt.37	91.222	1528	1923

**Table A4:** Statistics of genome annotation and comparative analysis of closely related *B. toyonensis* isolates Nt.18 and Nt.37.

Parameter	Isolate	
	Nt.18	Nt.37
Annotation statistics		
Features	5853	5905
Coding gene	5761	5814
RNAs	92	91
Distinct functions	4049	4072
Non-hypothetical proteins	3707	3729
Hypothetical proteins	2054	2085
Comparative analysis statistics		
Genes in core homolog families	5576	5594
Genes in singletons	185 (60) ^a^	220 (20) ^a^
Genes with ≥90% identity	5425	
Genes with <90% identity	151 (52) ^a^	

^a^ after hypothetical, unknown, phage and mobile element related features have been removed.

**Table A5:** Annotation results for secondary metabolite biosynthetic gene clusters No. 36 and 20 related to LAP and EPS biosynthesis, respectively, identified using the antiSMASH server. Feature number are indicated as in Supplementary Materials Tables S1 and S2.

Symbol	Genome Feature No.		Gene Name	Id., %	Pfam		
	Nt.18	Nt.37			Domain	Reference	E Value
Cluster number 36; Cluster type LAP							
Core biosynthetic genes							
HP	5593		Hypothetical protein		YcaO	PF02624.16	4 × 10^−58^
HP	5594		Hypothetical protein		Nitroreductase	PF00881.24	5.3 × 10^−11^
Cluster number 20; Cluster Type: saccharide, EPS related cluster							
Core biosynthetic genes							
UDPGE	3491	2631	UDP-glucose 4-epimerase (EC 5.1.3.2)	100	Epimerase	PF01370.21	1.5 × 10^−57^
MAEP	3503		Multi antimicrobial extrusion protein (Na(+)/drug antiporter), MATE family of MDR efflux pumps		Polysacc synt Polysacc synt C	PF01943.17 PF14667.6	9.6 × 10^−16^ 2 × 10^−9^
HP	3506		Hypothetical protein		Glycos transf 1 Glyco transf 4	PF00534.20 PF13439.6	9.1 × 10^−28^ 8.6 × 10^−9^
UFS	3508		UDP-N-acetyl-L-fucosamine synthase (EC 5.1.3.28)		Epimerase 2	PF02350.19	1.9 × 10^−88^
CPBP	3509		Capsular polysaccharide synthesis enzyme Cap5F		Epimerase GPI	PF01370.21 PF06560.11	4.4 × 10^−20^ 4.7 × 10^−5^
UGADH	3510		UDP-N-acetylglucosamine 4,6-dehydratase (EC 4.2.1.135)		Polysacc synt 2 Polysacc syn 2C	PF02719.15 PF08485.10	3 × 10^−103^ 9.2 × 10^−23^
CPBP	3511		Capsular polysaccharide biosynthesis protein Cps4F		Glycos transf 1 Glyco trans 4 4	PF00534.20 PF13579.6	3.2 × 10^−18^ 5.4 × 10^−13^
UPGPT	3512	2613	Undecaprenyl-phosphate galactosephosphotransferase (EC2.7.8.6)	40	Bac transf	PF02397.16	2.1 × 10^−62^
PPBP	3513		Probable polysaccharide biosynthesis protein EpsC		Polysacc synt 2 CoA binding 3	PF02719.15 PF13727.6	7 × 10^−124^ 6 × 10^−20^
UGP	3514	2612	UTP--glucose-1-phosphate uridylyltransferase (EC 2.7.7.9)	96	NTP transferase	PF00483.23	2.2 × 10^−35^
HP		2615	hypothetical protein	19	Glycos transf 1	PF00534.20	2.1 × 10^−13^
HP		2616	Hypothetical protein		Polysacc synt	PF01943.17	4.5 × 10^−15^
GTF		2620	Glycosyltransferase	22	Glyco trans 1 4	PF13692.6	3 × 10^−13^
EPGTF		2621	Exopolysaccharide biosynthesis glycosyltransferase EpsF (EC 2.4.1.)		Glyco transf 4 Glycos transf 1	PF13439.6 PF00534.20	1.4 × 10^−19^ 1.6 × 10^−24^
GTF		2622	Glycosyltransferase		Glyco transf 4 Glycos transf 1	PF13439.6 PF00534.20	1.3 × 10^−8^ 3.2 × 10^−26^
SPGT		2623	Sugar-phosphate guanylyltransferase/Sugar-phosephate isomerase		NTP transferase MannoseP isomer	PF00483.23 PF01050.18	2 × 10^−32^ 2.3 × 10^−19^
Additional biosynthetic and transport-related genes							
CPCT	3494	2628	Choline-phosphate cytidylyltransferase (EC 2.7.7.15)/Choline kinase (EC 2.7.1.32)	99	HTH 24 NTP transf 3 Choline kinase	PF13412.6 PF12804.7 PF01633.20	1.3 × 10^−11^ 3.5 × 10^−9^ 9.5 × 10^−34^
UMDH		2614	UDP-N-acetyl-D-mannosamine dehydrogenase (EC 1.1.1.336)		UDPG MGDP dh N UDPG MGDP dh UDPG MGDP dh C	PF03721.14 PF00984.19 PF03720.15	5 × 10^−58^ 5.8 × 10^−28^ 8.1 × 10^−20^
HP		2618	Hypothetical protein		Acyl transf 3	PF01757.22	1.3 × 10^−31^
HP	3493	2629	Hypothetical protein	100	EamA	PF00892.20	9.3 × 10^−12^
Other genes							
HP	3507		Hypothetical protein				
HP	3505		Hypothetical protein		O-ag pol Wzy	PF14296.6	4.7 × 10^−23^
HP	3504		Hypothetical protein		MAF flag10	PF01973.18	6.7 × 10^−14^
KDSB	3502		3-deoxy-manno-octulosonate cytidylyltransferase (EC 2.7.7.38)		CTP transf 3	PF02348.19	2.9 × 10^−55^
KDOPS	3501		2-Keto-3-deoxy-D-manno-octulosonate-8-phosphate synthase (EC 2.5.1.55)		DAHP synth 1	PF00793.20	1 × 10^−71^
API	3500		D-arabinose-5-phosphate isomerase (EC 5.3.1.13)		SIS CBS	PF01380.22 PF00571.28	1.1 × 10^−28^ 1.3 × 10^−8^
KDOPP	3499		3-deoxy-D-manno-octulosonate 8-phosphate phosphatase (EC 3.1.3.45)		Hydrolase 3	PF08282.12	2.6 × 10^−9^
HP	3498		Hypothetical protein		HIT	PF01230.23	3.1 × 10^−7^
LCP	3497	2625	Cell envelope-associated transcriptional attenuator LytR-CpsA-Psr, subfamily F2	75	LytR cpsA psr	PF03816.14	2 × 10^−50^
EPSX	3496	2626	EPSX protein	99			
HP	3495	2627	Hypothetical protein	100	LicD	PF04991.13	3.5 × 10^−6^
CPCT	3492	2630	Choline-phosphate cytidylyltransferase (EC 2.7.7.15)	98	NTP transferase	PF00483.23	1.8 × 10^−12^
HP		2617	Hypothetical protein		Hepar II III N Hepar II III	PF16889.5 PF07940.13	3.2 × 10^−16^ 5.4 × 10^−21^
HP		2619	Hypothetical protein				
CPCT		2624	Mannose-6-phosphate isomerase (EC 5.3.1.8)		PMI typeI	PF01238.21	5.8 × 10^−47^

Abbreviations: Id.—amino acid sequence identity of the Nt.18 and Nt.37 isolate protein homologs.
